# Elevational trends in hydraulic efficiency and safety of *Pinus cembra* roots

**DOI:** 10.1007/s00442-015-3513-1

**Published:** 2015-12-17

**Authors:** Adriano Losso, Andrea Nardini, Markus Nolf, Stefan Mayr

**Affiliations:** Department of Botany, University of Innsbruck, Sternwartestr. 15, 6020 Innsbruck, Austria; Dipartimento di Scienze della Vita, Università di Trieste, Via L. Giorgieri 10, 34127 Trieste, Italy

**Keywords:** Alpine timberline, Hydraulic conductance, Conifer, Root hydraulics, Xylem anatomy

## Abstract

In alpine regions, elevational gradients in environmental parameters are reflected by structural and functional changes in plant traits. Elevational changes in plant water relations have also been demonstrated, but comparable information on root hydraulics is generally lacking. We analyzed the hydraulic efficiency (specific hydraulic conductivity *k*_s_, entire root system conductance *K*_R_) and vulnerability to drought-induced embolism (water potential at 50 % loss of conductivity *Ψ*_50_) of the roots of *Pinus cembra* trees growing along an elevational transect of 600 m. Hydraulic parameters of the roots were compared with those of the stem and related to anatomical traits {mean conduit diameter (*d*), wall reinforcement [(*t/b*)^2^]}. We hypothesized that temperature-related restrictions in root function would cause a progressive limitation of hydraulic efficiency and safety with increasing elevation. We found that both root *k*_s_ and *K*_R_ decreased from low (1600 m a.s.l.: *k*_s_ 5.6 ± 0.7 kg m^−1^ s^−1^ MPa^−1^, *K*_R_ 0.049 ± 0.005 kg m^−2^ s ^−1^ MPa^−1^) to high elevation (2100 m a.s.l.: *k*_s_ 4.2 ± 0.6 kg m^−1^ s^−1^ MPa^−1^, *K*_R_ 0.035 ± 0.006 kg m^−2^ s^−1^ MPa^−1^), with small trees showing higher *K*_R_ than large trees. *k*_s_ was higher in roots than in stems (0.5 ± 0.05 kg m^−1^s^−1^MPa^−1^). *Ψ*_50_ values were similar across elevations and overall less negative in roots (*Ψ*_50_ −3.6 ± 0.1 MPa) than in stems (*Ψ*_50_ −3.9 ± 0.1 MPa). In roots, large-diameter tracheids were lacking at high elevation and (*t/b*)^2^ increased, while *d* did not change. The elevational decrease in root hydraulic efficiency reflects a limitation in timberline tree hydraulics. In contrast, hydraulic safety was similar across elevations, indicating that avoidance of hydraulic failure is important for timberline trees. As hydraulic patterns can only partly be explained by the anatomical parameters studied, limitations and/or adaptations at the pit level are likely.

## Introduction

In alpine regions, the obvious change in vegetation with increasing elevation corresponds to changes in structural and functional plant traits, including plant hydraulics. This is particularly relevant for trees as their crown is in close contact with the atmosphere, and water has to be transported over long distances within the tree (Mayr 2007).

According to the cohesion–tension theory, water is transported in the xylem under tension. In this metastable state (Tyree and Zimmermann 2002), even a small perturbation of the system can lead a sudden transition from the liquid to the vapor phase (Tyree and Sperry 1989; Steudle 2001), resulting in xylem embolism (e.g., Nardini et al. 2011). Drought and freeze–thaw stress are known to be the main factors inducing xylem embolism. The vulnerability to embolism is species-specific (Jacobsen et al. 2007; Choat et al. 2008, 2012; Tixier et al. 2014), but there may also be some intra-specific variability as shown, for example, by Delzon et al. (2010) for conifers. Furthermore, several studies have reported that vulnerability to embolism differs between plant organs, in particular between roots and shoots (Sperry and Saliendra 1994; Alder et al. 1996; Sperry and Ikeda 1997; Tsuda and Tyree 1997; Kavanagh et al. 1999; Hacke et al. 2000; Cochard et al. 2002; Martinez-Vilalta et al. 2002; Froux et al. 2005). Analyses of plant traits related to hydraulic safety (i.e., vulnerability to embolism) and hydraulic efficiency (i.e., hydraulic conductivity/conductance) should take into careful consideration this variability within plants and individuals.

Alpine ecosystems are characterized by substantial elevational changes in environmental parameters, among which the progressive decrease in temperature at increasing elevation is the most relevant for plants (Körner 2003). Temperature also plays an important role from a hydraulic point of view: low temperatures cause an increase in water viscosity (e.g., about 2.4 % per kelvin degree; Tyree and Zimmermann 2002), thus affecting transport velocities in plants (Sellin and Kupper 2007). When temperatures reach the freezing point, the water supply of plants breaks down (Burke et al. 1986; Sakai and Larcher 1987), and frost–drought and freeze–thaw events can lead to xylem embolism (Groß et al. 1991; Sperry and Sullivan 1992; Mayr et al. 2003a, b; Mayr and Zublasing 2010). Low temperatures may also affect plant hydraulics at the cellular level, limiting growth processes and thus the formation of required transport structures (Boyce and Saunders 2000; Körner 2003; Alvarez-Uria and Körner 2007) or influencing specific cell functions, such as stomata regulation. Several authors have reported that changes in xylem anatomical traits determine the cavitation resistance (e.g., Hacke et al. 2004; Domec et al. 2006). Petit et al. (2011) demonstrated that at high elevation, xylogenesis is limited by low temperature, which in turn causes reduced hydraulic efficiency and hence limits longitudinal growth of *Picea abies* (see also Rossi et al. 2007).

Körner (1998) hypothesized that low temperatures limit tree growth and survival. Below critical temperature thresholds, basic metabolic processes may not reach the minimum rate required for growth and tissue renewal but still allow mere survival. The low temperatures and resulting cold soils also affect root growth and function (Tranquillini 1979; Alvarez-Uria and Körner 2007). Roots represent an important part of the plant hydraulic pathway and, consequently, affects shoot functioning (Havranek 1972; Scott et al. 1987). Havranek (1972) reported a drop in daily photosynthetic rates when the root zone temperature of *Pinus cembra* was between 0 and 7 °C. Exposure of plants to low temperatures has been demonstrated to considerably increase membrane hydraulic resistance (Lee et al. 2004), which contribute 10–25 % of the overall cellular water conductivity (Steudle and Henzler 1995; Ye et al. 2003).

Several studies have investigated root functioning and its linkages to whole plant physiology (Tranquillini 1973, 1979; Goldstein et al. 1985; Häsler et al. 1999), but to our knowledge, the effects of elevational changes on root hydraulics are a little explored aspect of plant physiology. In the study reported here, we analyzed the root hydraulics of *Pinus cembra* along an elevational transect extending over 600 m, focusing on aspects of hydraulic efficiency [specific hydraulic conductivity (*k*_s_) and entire root system conductance (*K*_R_)] and hydraulic safety (vulnerability to drought-induced cavitation) of young trees. These parameters were compared with stem hydraulic traits and related to anatomical features (conduit diameter, wall reinforcement, mean diameter of conduits, together accounting for 95 % of hydraulic conductivity). We hypothesized that hydraulic efficiency and safety would be impaired at higher elevation due to elevational changes in environmental parameters and, consequently, that tree life would be limited. Limitations in root hydraulic parameters thereby should be correlated with xylem anatomical parameters.

## Materials and methods

### Plant material and sampling

Measurements were performed on the roots and stems of stone pine (*Pinus cembra* L.) trees. *P. cembra* is a conifer which is found widespread throughout the subalpine zone of the Central Alps and Carpatian Mountains, either in mixed or pure stands. It typically occurs at 1200–2200 m a.s.l. although it occasionally reaches higher elevations (highest documented stand: 2390 m a.s.l. in Engadin, Switzerland; Mattes 1982). It is one of the few tree species found at the upper elevational limits of tree distribution.

We selected trees growing along a 600-m elevational transect, beginning at 1500 m a.s.l. and extending up to 2100 m a.s.l. The study site was located near Praxmar (1700 m a.s.l., 47°09′N/11°07′E) in the Tyrolean Central Alps (Austria). The selected transect was south-east exposed except for the lowest part (1500–1600 m a.s.l.) which was exposed to the south-west.

Eleven sites at different elevations along the transect were chosen for the analysis of root *k*_s_ (see section “Root xylem hydraulic conductivity”), and from each site, we collected about ten roots (diameter 0.5–1 cm) between April and May 2013. These roots were carefully excavated in the upper 20 cm of soil, cut to lengths of 20–30 cm, immediately re-cut under water, and then wrapped in plastic bags before being transported to the laboratory.

Vulnerability analysis was performed on about 25 roots and 25 stems collected between June and July 2013 at three different elevations (1500, 1750, and 2100 m a.s.l.) along the transect. At each elevation, roots were collected as for measurements of hydraulic conductivity (see above). In addition, sun-exposed branches were harvested on 3- to 4-m-tall trees at the selected sites, re-cut under water, and then transported to the laboratory while maintained in a bucket filled with water and covered with a plastic bag.

Plants for the analysis of *K*_R_ (see section “Root system hydraulic conductance”) were chosen at three elevational ranges (about 1550, 1800 and 2050 m a.s.l.). Due to the time-consuming nature of these measurements, only three *P. cembra* individuals with similar characteristics (height approx. 200 cm, similar crown size, single-standing trees, sites with similar slope and exposure) were selected per elevation. For each tree, tree height, length of the longest sun-exposed and of the longest shaded twig, respectively, distance of the crown base to the soil, and xylem cross-sectional area at the level at which the trunk base was cut were recorded to estimate crown size. Another set of *K*_R_ measurements was made on seven specimens of different size (range in height 42–211 cm) found at elevations ranging from 2000 to 2100 m a.s.l. to check for tree height effect. All *K*_R_ measurements were performed in August 2013.

### Root xylem hydraulic conductivity

Hydraulic measurements were performed using a modified Sperry apparatus (Sperry et al. 1988; Chiu and Ewers 1993; Vogt 2001) as described by Mayr et al. (2006). Specifically, roots were immersed in distilled water, the bark was removed, and samples were re-cut several times with a sharp wood carving knife to gradually release tension (Wheeler et al. 2013) and obtain segments about 5–6 cm long, with diameters of between 3 and 6 mm. An infusion bag was filled with distilled, filtered (pore size 0.22 µm), and degassed water containing 0.005 % (v/v) “Micropur” (Katadyn Products, Wallisellen, Switzerland) to prevent microbial growth and connected to the hydraulic system. Samples were connected to a fivefold valve (Luer-lock system; neoLab Migge Laborbedarf-Verttriebs GmbH, Heidelberg, Germany). The flow rate was determined on about ten roots per site (see section “Plant material and sampling”) either by recording root weight every 10 s on a PC-connected balance (Sartorius BP61S; precision 0.1 mg; Sartorius AG, Göttingen, Germany) and fitting a linear regression over 200-s intervals, or using the Xylem Embolism Meter (XYL’EM system; Bronkhorst, Montigny-les-Cormeilles, France; Cochard et al. 2000). Measurement pressure was set to 0.004 MPa. All root segments were flushed for 15–30 min at 0.008 MPa, and flushing was repeated until the measurements showed no further increase in conductivity. The maximum conductivity was used to calculate the specific hydraulic conductivity (*k*_s_) normalized by the xylem cross-sectional area. Hydraulic conductivity was measured on 8–15 samples per elevation.

### Root system hydraulic conductance

Root system hydraulic conductance was measured using the high-pressure flow meter (HPFM Gen 3; Dynamax, Houston, TX; Tyree et al. 1995). For these measurements, the stem base of selected trees (see section “Plant material and sampling”) was cut at 10–20 cm above the soil surface, and the excised root system was placed immediately after cutting into a receptacle made out of plastic bags and tape (mounted before cutting) filled with water in order to keep the cut section at the base level of the excised root system immersed in water. The cut section was re-cut several times under water with a carving knife to remove potentially introduced embolism. The first cut was done in air because xylem tracheids are short enough to allow a second cut under water without risk of air penetration into the root system. The base of the excised root system was connected to the HPFM flow meter and perfused with the same solution used for the root *k*_s_ measurements (see section “Root xylem hydraulic conductivity”). Three to five transient measurements (see Tyree et al. 1995) were immediately done and the hydraulic conductance (*K*; kg s^−1^ MPa^−1^) calculated. *K* was normalized by the stem cross-sectional area to calculate *K*_R_ (kg m^−2^ s^−1^ MPa^−1^). The stem cross-sectional area was chosen as reference parameter because (1) it is the area to which the HPFM was connected and (2) we expected this area would represent the supplied crown biomass as larger crowns will correspond to larger stem cross-sectional areas even when tree height is similar. This is an important characteristic of trees at the timberline where trees differ considerably in crown sizes due to damage (breakage) caused by snow and wind and subsequent dwarf growth.

Fitting of the plot *K*_R_ versus tree height (Fig. 2) was optimized by use of the inverse first-order plot.

### Vulnerability analyses

Hydraulic vulnerability was analysed on 23–29 stems and on 23–28 roots collected at 1500, 1750 and 2100 m a.s.l. (Table 1). Each stem was harvested from a different tree. Roots were excavated at different sites, but due to the long roots of *P. cembra* and to the rocky underground, root samples could not always be clearly assigned to a specific nearby standing tree. Vulnerability curves were obtained by a two-step procedure. Negative *Ψ* were first induced in the samples by use of centrifugal force (Pockman et al. 1995; Alder et al. 1997), and then the percentage of loss of hydraulic conductance (PLC) was measured with the XYL’EM system (Xylem Embolism Meter; Bronkhorst, Montigny-les-Cormeilles, France; Cochard et al. 2000). Root and stem segments (length 15 cm) were debarked at the ends under water. Segments were positioned in the centrifuge and fixed via thin aluminum plates secured by screws inside a 150-mm rotor in a Sorvall RC-5 centrifuge (Thermo Fisher Scientific, Waltham, MA). Sample ends were kept inside ‘L’-shaped water reservoirs fitted in slots within the rotor. The use of these reservoirs allowed us to keep all conduits filled with water during centrifugation (Alder et al. 1997). A maximum of three segments were spun at once. Equation 1 (Beikircher et al. 2010) was used to calculate the spinning velocity necessary to impose the desired water potential at the segment’s center:

1 $${\text{RPM }} = \, \left( {\varPsi / \, \left( { 5. 4 8\; \times \; 10^{ - 6} \; \times \;r^{ 2} } \right)} \right)^{0. 5}$$

where RPM is rotations per minute, *Ψ* is the water potential (MPa), and *r* is the radius of the rotor (m). We imposed six negative *Ψ* values, i.e., −1, −2, −3, −4, −5 and −6 MPa, respectively. For every *Ψ* level, at least three replicates were measured. After spinning, segments were re-cut and debarked under water to obtain 5- to 8-cm-long root segments and 4- to 6-cm-long stem segments from the samples’ center. The diameter of the debarked root and stem samples was between 4 and 7 mm and between 5 and 8 mm, respectively. PLC was determined using the XYL’EM system by measuring the increase in hydraulic conductivity after removal of xylem embolism by repeated high-pressure flushes (Sperry et al. 1988). Flushing (at 0.1 MPa for 15–20 min) and conductivity measurements were performed with water prepared as for root xylem hydraulic conductivity (see section “Root xylem hydraulic conductivity”). Flushing was repeated until measurements indicated no further increase in conductivity. PLC was calculated as:

2 $${\text{PLC }} = { 1}00 \, \left( { 1- k_{\text{i}} /k_{\text{f}} } \right)$$

where *k*_I_ and *k*_f_ are the initial and the final conductivity, respectively. Curves were fitted using an exponential sigmoidal equation (Eq. 3), according to Pammenter and Vander Willingen (1998):

3 $${\text{PLC }} = { 1}00/ \, \left( { 1 { } + { \exp }\left( {a\left( {\varPsi - \varPsi_{ 50} } \right)} \right)} \right)$$

where PLC is the percentage loss of conductivity, *Ψ* is the corresponding water potential (MPa), and coefficient *a* is related to the slope of the curve. *Ψ*_50_ is the *Ψ* value corresponding to 50 % loss of conductivity and is located in the steepest part of the curve, where even small changes in xylem tension induce a large decline in conductivity. We also calculated the water potentials at 12 % (*Ψ*_12_) and at 88 % (*Ψ*_88_) loss of conductivity. *Ψ*_12_ is an estimate of the xylem pressure at which embolism begins, and *Ψ*_88_ is an estimate of the xylem pressure at critical embolism level (Sparks and Black 1999; Domec and Gartner 2001; Choat et al. 2012).

**Table 1:** Hydraulic efficiency in roots and stems harvested at different elevations

Elevation (m a.s.l.)	Water potential (*Ψ*) (MPa)^a^			Parameter *a* ^b^	Regression coefficient (*r* ^2^)	Number of samples (*n*)
	*Ψ*_50_	*Ψ*_12_	*Ψ*_88_			
Roots						
2100	−3.71 ± 0.10 a	−2.02 ± 0.28 a	−5.39 ± 0.08 ab	1.18 ± 0.13 a	0.950	25
1750	−3.51 ± 0.12 a	−1.84 ± 0.37* a	−5.19 ± 0.13* a	1.19 ± 0.17* a	0.897	28
1500	−3.60 ± 0.14* a	−1.55 ± 0.39 a	−5.66 ± 0.12 b	0.97 ± 0.12 a	0.928	23
Stems						
2100	−3.94 ± 0.09 ab	−2.38 ± 0.26 a	−5.51 ± 0.08 a	1.27 ± 0.14 a	0.957	23
1750	−3.73 ± 0.07 a	−2.95 ± 0.19 a	−4.50 ± 0.05 b	2.56 ± 0.40 b	0.973	23
1500	−4.18 ± 0.14 b	−2.40 ± 0.39 a	−5.96 ± 0.12 c	1.12 ± 0.16 a	0.912	29

Values are presented as the mean ± standard error (SE) unless indicated otherwise. Within each set of plant organs, values within one column not followed by the same letter differ significantly at *P* < 0.05 (Student’s *t* test)

* Significant difference between roots and stems at *P* < 0.05

^a^ *Ψ*_50_, *Ψ*_12_, *Ψ*_88_, *Ψ* value corresponding to 50, 12, and 88 %, respectively, loss of conductivity

^b^ *a* is a coefficient related to the slope of the vulnerability curve

### Anatomical analysis

Anatomical analyses were performed on randomly selected samples (10 stems and 12 roots per elevation) collected from trees of similar height (3–4 m) which had been previously used for the vulnerability analysis. Petit et al. (2009) stated that tree height might be the most relevant factor for conduit tapering and reported similar values among plants of different age but similar height. Cross-sections were cut with a microtome (Sledge Microtome G.S.L. 1; Schenkung Dapples, Zurich, Switzerland) and stained with phloroglucinol-HCl (to stain lignin in red). Anatomical parameters were analyzed on images captured with a light microscope (Olympus BX41; Olympus Austria, Vienna, Austria) connected to a digital camera (Cybershot DSC-W17; Sony Corp. Tokyo, Japan). Images were analyzed using Image J 1.37 software (National Institute of Health, Bethesda, MD).

To analyze a representative area within the sapwood, we measured individual conduit lumen areas in radial sectors of the youngest three annual rings (formed in 2011–2013). Between 700 and 1500 tracheid areas per sample were measured. A square shape was assumed, and the square root of the area was calculated for each tracheid to determine the tracheid diameter. For each sample, the mean, maximum, and minimum tracheid diameters were calculated (*d*_mean_, *d*_max_, and *d*_min_, respectively). The mean hydraulic conduit diameter (*d*_h_) was calculated according to Eq. 4 (Sperry and Hacke 2004):

4 $$d_{\text{h}} = \sum d^{ 5} /\sum d^{ 4}$$

where *d* is the diameter (micrometers) of the analyzed tracheid.

In order to quantify conduit wall reinforcement, the ‘thickness-to-span ratio’ (*t/b*)^2^, which is related to cavitation resistance and wall collapse (Hacke et al. 2001), was determined for at least eight tracheid pairs per sample in seven to eight samples per elevation. The wall thickness (*t*) and the lumen breadth (*b*) were directly measured using Image J software. Measurements were made on tracheid pairs, whereby the analyzed tracheids showed a diameter similar to *d*_h_ ± 3 μm for roots and ±2 μm for stems (see also Hacke and Sperry 2001; Hacke et al. 2001).

The mean diameter of all conduits accounting for 95 % of hydraulic conductivity of a root or a stem (D95; Tyree et al. 1994) was calculated to quantify the relative contribution of conduits of different size to the overall flow. The diameters of all tracheids measured were subdivided into diametric classes. The fourth power of the mean diameter of each class was multiplied by the tracheid number contained in each class, and the number of tracheids in each class was divided by the total number of tracheids, thus representing the contribution of each single diameter class to the total hydraulic flow. Contribution percentages, starting from the highest contributions, were summed one by one, until the sum reached 95 % of the total conductance. Therefore, the diameter classes used for further analysis contributed 95 % to the overall hydraulic conductivity.

### Statistical analysis

All values are given as mean ± standard error. Differences were tested using a two-way analysis of variance followed by Tukey’s post hoc comparison (*K*_R_, anatomical parameters) or by the Student’s *t* test, after testing for normal distribution and homoscedasticity (*k*_s_; roots vs. stems, vulnerability analyses). Vulnerability curves were constructed from cumulative PLC measurements of several samples so that statistical analyses had to be based on the entire curve, and differences were tested best with Student´s *t* test. Correlation analysis was carried out using the Pearson product–moment correlation [*k*_s_; *K*_R_ vs. elevation; (*t/b*)^2^ vs. elevation; *K*_R_ vs. tree height]. All tests were conducted using SPSS software version 21.0 (SPSS Inc., Chicago, IL) at a probability level of 5 %.

## Results

### Root xylem hydraulic conductivity

Mean values of *k*_s_ were significantly higher in roots (5.07 ± 0.29 kg m^−1^ s^−1^ MPa^−1^) than in stems (0.53 ± 0.05 kg m^−1^ s^−1^ MPa^−1^; *P* < 0.001). Root *k*_s_ values were not significantly different before and after flushing, suggesting low levels of native embolism in the samples.

**Fig. 1:**
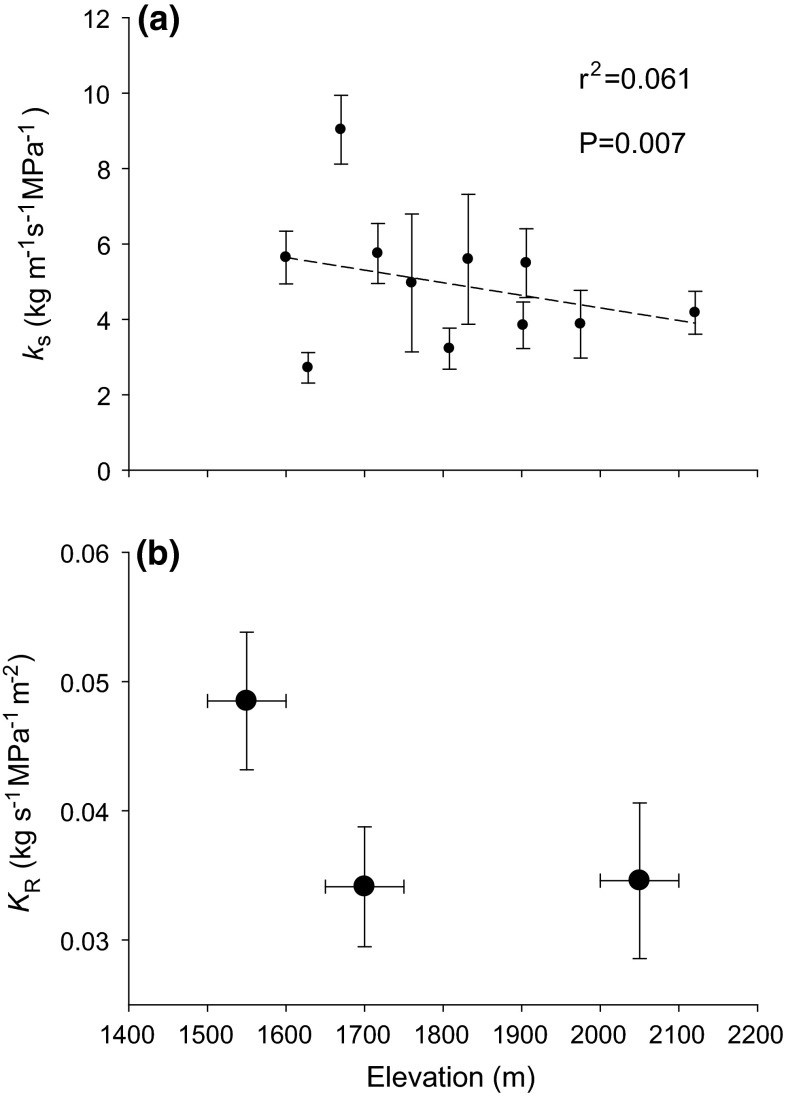
**a** Root-specific hydraulic conductivity (*k*_*s*_; g m^−1^ s^−1^ MPa^−1^) vs. elevation. *Dashed line* Trend between *k*_s_ and elevation. For clarity, mean ± standard error per elevation is shown, while individual *k*_s_ values were used for statistics (*n* = 118).** b** Root system hydraulic conductance (*K*_*R*_; kg m^−2^ s^−1^ MPa^−1^) vs. elevation (mean ± SE)

Figure 1a indicates a trend of decreasing root *k*_s_ with increasing elevation, albeit variation in *k*_s_ along the elevational transect was high.

### Root system hydraulic conductance

Root system hydraulic conductance was not significantly different across elevations. However, Fig. 1b indicates a trend for lower *K*_R_ at higher elevations: a *K*_R_ value of 0.0485 ± 0.0053 kg m^−2^ s^−1^ MPa^−1^ was recorded at the lowest elevation (1500–1600 m a.s.l.), decreasing to 0.0341 ± 0.0046 and 0.0346 ± 0.0060 kg m^−2^ s^−1^ MPa^−1^ at 1750–1850 and 2000–2100 m a.s.l., respectively.

**Fig. 2:**
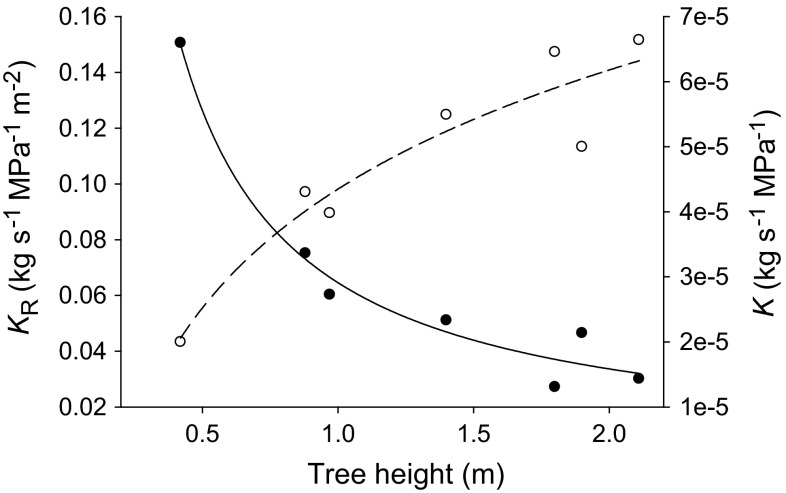
Root system hydraulic conductance (*K*_*R*_: kg m^−2^ s^−1^ MPa^−1^; *filled circles*, *solid line*) and root absolute hydraulic conductance (*K*; kg s^−1^ MPa^−1^; *open circles*, *dashed line*) vs. tree height of trees growing at the timberline. *K*_R_: *r* ^2^ = 0.97, *P* = 0.01; *K*: *r* ^2^ = 0.89, *P* = 0.004

The comparison of *P. cembra* trees of different height (at 2100 m a.s.l.) revealed a significant correlation (*P* = 0.01) between tree height and *K*_R_ (Fig. 2). The smallest tree (height 0.42 m) showed the highest *K*_R_ (0.1506 kg m^−2^ s^−1^ MPa^−1^), which indicates that changes in *K*_R_ are most pronounced during the development of young trees. A similar negative correlation was observed when measured conductances (*K*) were normalized by tree height instead by stem cross-sectional area (data not shown).

### Vulnerability analysis

**Fig. 3:**
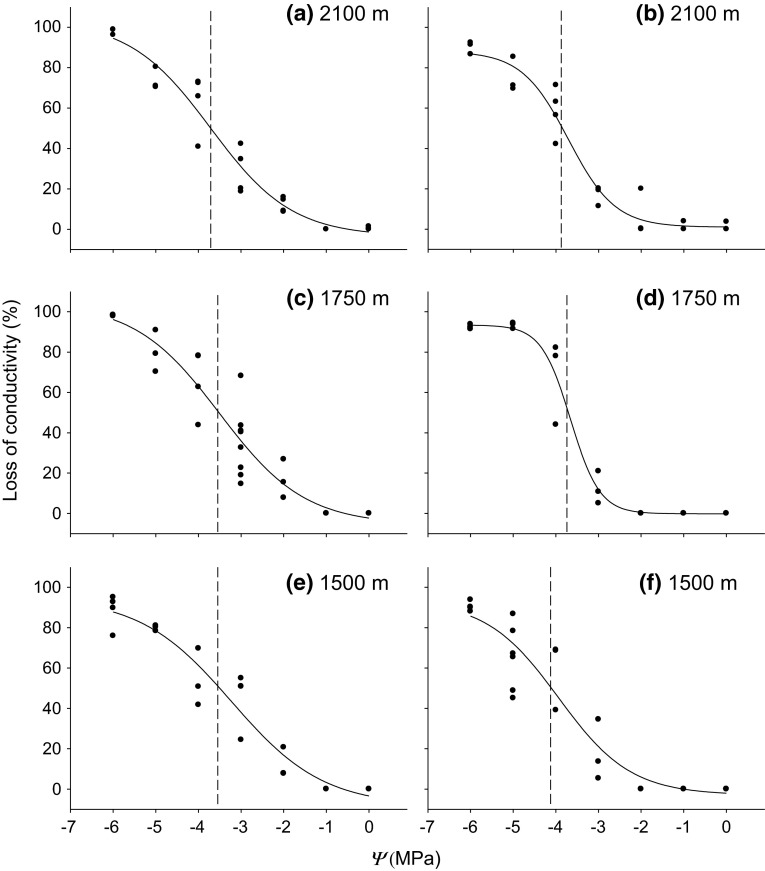
Vulnerability to drought-induced embolism of roots (**a**, **c**, **e**) and stems (**b**, **d**, **f**) harvested at different elevations. Percentage loss of conductivity (PLC) was plotted versus water potential (*Ψ*) and the curves fitted to a sigmoid function according to Pammenter and Vander Willingen (1998; see “Materials and methods”). *Dashed vertical lines* *Ψ*_50_ (water potential at 50 % loss of conductivity)

Vulnerability curves of roots (Fig. 3) were sigmoidal and showed similar values of *Ψ*_50_ (Pammenter and Vander Willingen 1998) at all elevations (Table 1). The most negative root *Ψ*_50_ was observed at the highest elevation (−3.71 ± 0.10 MPa; Table 1). Similarly, there was a decrease in water potential at 12 % loss of conductivity (*Ψ*_12_) with increasing elevation, while the coefficient *a* (related to the slope of the curve; Pammenter and Vander Willingen 1998) increased along the same gradient (Table 1). The water potential at 88 % loss of conductivity (*Ψ*_88_) was most negative at 1500 m a.s.l. (−5.66 ± 0.12 MPa; Table 1).

Compared to stems, in roots the *Ψ*_50_ values were overall less negative and the coefficient *a* values higher. The *Ψ*_12_ and *Ψ*_88_ values were also less negative in roots than in stems, except for *Ψ*_12_ at 1500 m a.s.l. and *Ψ*_88_ at 1750 m a.s.l. (Table 1). Both roots and stems showed relatively flat curves at the lowest elevation.

### Anatomical analysis

**Fig. 4:**
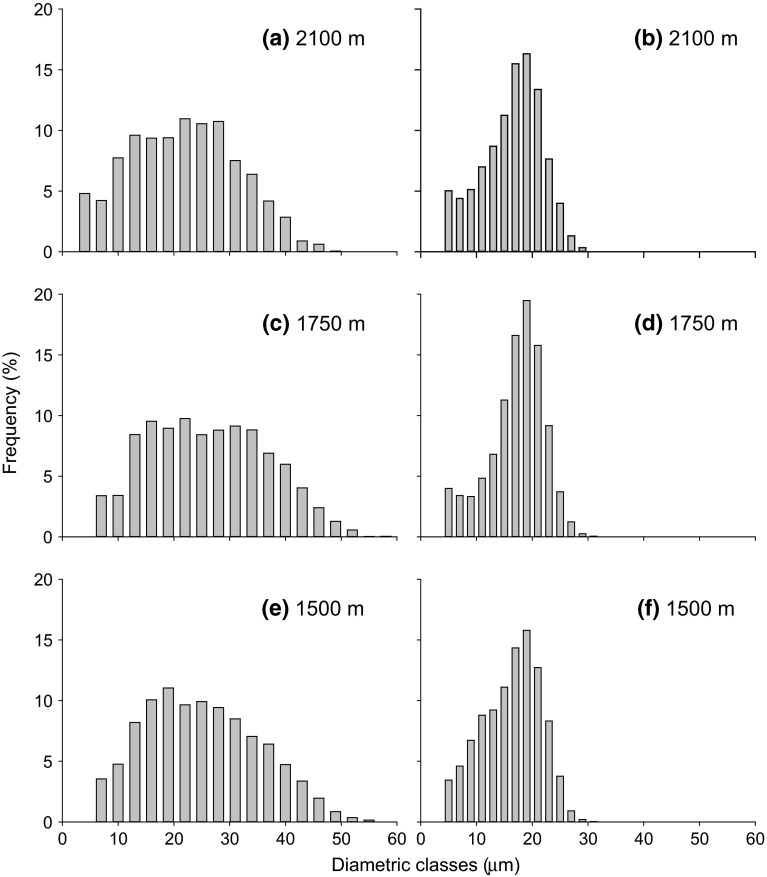
Distribution of tracheid diameters (3-µm classes for roots and 2-µm classes for stems) in roots (**a**, **c**, **e**) and stems (**b**, **d**, **f**) harvested at different elevations. All tracheids with a diameter of >5 µm were included

According to Fig. 4, roots at the highest elevation (2100 m a.s.l.) had smaller tracheids than those at the other two elevations, based on the observation that at the highest elevation larger diameter classes (>50 μm) were less frequent and small diameter classes (<5 µm) were more frequent (see Fig. 4a). Mean hydraulic diameters (*d*_h_) of root tracheids were similar across the elevation transect (Table 2; Fig. 4), with slightly higher *d*_h_ (34.44 ± 1.40 μm) at 1750 m a.s.l. than at the other two elevations (32.23 ± 1.42 μm at 1500 m a.s.l. and 32.34 ± 1.04 μm at 2100 m a.s.l., respectively). Also in stems, no significant differences in *d*_h_ across elevations were observed (Table 2). *d*_h_ was overall higher in roots than in stems (*P* < 0.001). The mean diameter of conduits accounting for 95 % of hydraulic conductivity (D95; Tyree et al. 1994) was not significantly different in roots and stems across elevations (see Table 2), but the D95 of roots was higher than that of stems (*P* = 0.003; see Table 2).

**Table 2:** Anatomical parameters of roots and stems harvested at different elevations

Elevation (m a.s.l.)	Anatomical parameters^a^				Number of samples (*n*)
	*d* (μm)	*d*_h_ (μm)	(*t/b*)^2^	D95 (μm)	
Roots					
2100	22.27 ± 0.93* a	32.34 ± 1.42* a	0.022 ± 0.001* a	29.0	13
1750	23.73 ± 1.03* a	34.44 ± 1.40* a	0.030 ± 0.002* ab	30.8	12
1500	22.60 ± 1.23* a	32.23 ± 1.04* a	0.041 ± 0.007* b	28.8	14
Stems					
2100	14.40 ± 0.28 a	19.02 ± 0.39 a	0.192 ± 0.015 a	17	10
1750	15.18 ± 0.23 a	19.09 ± 0.28 a	0.204 ± 0.010 a	17	10
1500	14.26 ± 0.17 a	19.07 ± 0.21 a	0.126 ± 0.036 b	17	9

Values are presented as the mean ± SE. Within each set of plant organs, values within one column not followed by the same letter differ significantly at *P* < 0.05 (two-way analysis of variance)

* Significant difference between roots and stems at *P* < 0.05

^a^Mean tracheid diameter (*d*), mean hydraulic diameter (*d*_h_), cell-wall reinforcement (*t/b*)^2^, and diameter of conduits accounting for 95 % of hydraulic conductivity (D95) of roots and stems harvested at different elevations

**Fig. 5:**
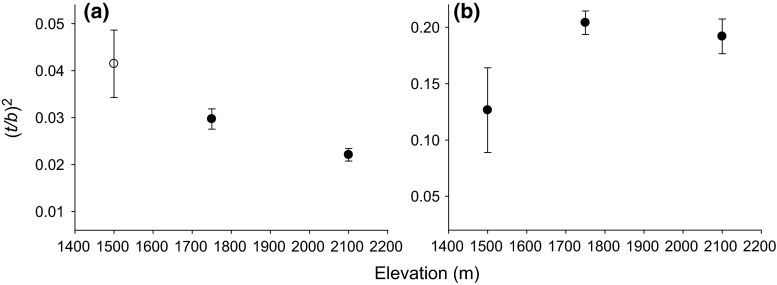
Cell-wall thickness-to-span ratio [(*t/b*)^*2*^] in roots (**a**) and stems (**b**) harvested at different elevations. For clarity, the mean ± SE per elevation are shown

Cell-wall reinforcement (*t/b*)^2^ significantly differed between roots and stems (*P* = 0.003; Fig. 5), with higher values in stems than in roots (*P* < 0.001). (*t/b*)^2^ was significantly correlated with elevation in both roots (*P* = 0.0125) and stems (*P* = 0.0139; all statistics based on individual values). In roots, (*t/b*)^2^ at the lowest elevation (0.04 ± 0.01) was twofold higher than that at 2100 m a.s.l. (see Table 2); in contrast, stem (*t/b*)^2^ increased with elevation (Fig. 5; Table 2).

## Discussion

In accordance with our working hypothesis, root hydraulic efficiency decreased with elevation. Both specific hydraulic conductivity (*k*_*s*_) and entire root system conductance (*K*_*R*_) were lowest at the highest study sites. In contrast and surprisingly, hydraulic safety, i.e., resistance to drought-induced embolism, was not influenced by elevation. Similar water potential at 50 % loss of conductivity (*Ψ*_50_) in all trees indicates that embolism avoidance is of comparable importance at all elevations. These elevational trends in root hydraulics are discussed in the following sections and related to the analyses conducted on branches, the anatomical analyses, and the role of the tree size.

### Hydraulic efficiency

Stone pine tracheids showed overall higher *k*_s_ in roots than in stems, corresponding to a lower *d* and *d*_h_ in the latter (Table 2). However, *k*_s_ also depends on the resistance of pit connections (Lancashire and Ennos 2002; Tyree and Zimmermann 2002), which was not analyzed in the present study. Variation in pit hydraulics (Mayr et al. 2002; Choat et al. 2008; Hacke and Jansen 2009; Schulte et al. 2015) may explain the observed root *k*_s_ trend of lower conductivities at higher elevation (Fig. 1). Even a small change in pore anatomy, such as a change in the area of one individual pore in the margo, can have a strong effect on the flow velocity (Schulte et al. 2015). In our study, the *d* and *d*_h_ of roots and stems were similar across elevations (Table 2), which confirms previous studies reporting that the tracheid size of *P. cembra* stems is similar at low and high elevations (600 and 2100 m a.s.l., respectively; Mayr et al. 2006). We also found that root and stem diameter of all conduits accounting for 95 % of hydraulic conductivity (D95; Tyree et al. 1994) were similar at the three elevations in both the roots and stems. Only tracheids with a width of >50 µm were less frequent at the highest elevation (2100 m a.s.l.; Fig. 4a). According to Pittermann and Sperry (2003, 2006), wider tracheids are more vulnerable to freezing-induced embolism, possibly implying that the decreased number of the widest tracheids at high elevations might be an adaptation to freezing stress, as also suggested by Mayr et al. (2003a). The absence of changes in tracheid dimensions across elevations indicates that xylogenesis was not significantly inhibited by low temperatures at the high elevation, as observed by Petit et al. (2011), whereby possible changes in the longitudinal increment of plant organs in our study were not taken into account.

The relation between the absolute conductance (*K*) of the entire root system and tree height of trees growing at the timberline showed a progressive increase of *K* with increasing tree height (filled dots, Fig. 2). This correlation fits data reported by Mencuccini (2002; see, for example, Fig. 3 therein), as well as the increase in the total plant hydraulic conductance with tree height reported by West et al. (1999) and Mencuccini (2002). We normalized *K* by the cross-sectional area of the cut section (*K*_R_) as an estimate for the crown biomass (see section “Materials and methods”) and obtained a negative correlation between tree height and *K*_R_ (filled circles, Fig. 2). This result suggests that small trees have comparably larger root systems or, alternatively, more efficient roots than big trees. A similar situation has been reported for *Quercus suber*, which develops a deep root system during the early stages of plant establishment (Tsakaldimi et al. 2005). *P. cembra* can also form deep and efficient root systems (Mattes 1982; Mayr 2007). Our data indicate that young *P. cembra* trees first invest in forming an efficient root system, and then, with reliable water and nutrient sources, growth of aboveground biomass is forced. In contrast, the observed trend toward lower *K*_R_ at higher elevation (Fig. 1b) suggests physiological and functional limitation of the entire root system that is characterized by, for example, changes in root length, root diameter, and/or area of fine roots and/or by tissue properties in the water uptake zone. It is important to note that the elevational trend in *K*_R_ observed in our study was not an effect of tree height as the trees compared were of similar height.

### Hydraulic safety

Several studies have compared vulnerability to embolism of stems and roots, with conflicting reports of more vulnerable root xylem in angiosperms (Sperry and Saliendra 1994; Alder et al. 1996; Tsuda and Tyree 1997; Martinez-Vilalta et al. 2002) and conifers (Sperry and Ikeda 1997; Kavanagh et al. 1999; Hacke et al. 2000) or more vulnerable shoot xylem in angiosperms (Cochard et al. 2002; Froux et al. 2005), with a study on *Pinus nigra* revealing no significant difference between the two plant organs (Froux et al. 2005). In the study presented here, *P. cembra* roots (Table 1) were overall more vulnerable to cavitation than stems. A higher vulnerability to embolism can be related to the presence of larger tracheids in roots, as reported for *Betula occidentalis* (Sperry and Saliendra 1994), *Cupressus sempervirens*, and *Pinus halepensis* (Froux et al. 2005). This was also observed in our samples, where root tracheids were approximately 1.5-fold wider than those of stems (Table 2; Fig. 4). Larger conduits would suffer a greater proportional loss of conductivity for each conduit lost to water transport (Choat et al. 2008), and they probably also tend to have a higher cumulative pit area than small ones; consequently, larger conduits likely have a higher probability of pit dysfunction (pit area hypothesis; Wheeler et al. 2005, Hacke et al. 2006; Sperry et al. 2007).

Mayr et al. (2003b, 2006) reported a significant difference of approximately 0.3 MPa in *Ψ*_50_ of *P. cembra* stems harvested at low (600 m a.s.l.) and high elevation (2100 m a.s.l.). The *Ψ*_50_ at the highest site of Mayr et al. (2003b, 2006) was slightly less negative than the *Ψ*_50_ obtained at 2100 m a.s.l. in our study (−3.94 ± 0.09 MPa; Table 1). We found no consistent trend in vulnerability thresholds in roots or stems on a transect between 1500 and 2100 m a.s.l. Only *Ψ*_88_ at the lowest elevation was remarkably negative in roots and stems (Table 1). Root *Ψ*_12_ decreased with increasing elevation, which might suggest the existence of plant adaptations to avoid early stages of embolism formation. Overall, *P. cembra* did not exhibit notable vulnerability adaptation at increasing elevation. This plant species is known to form an efficient cuticular shield which enables it to maintain shoot *Ψ* above −2 MPa for long periods when stomata are closed (Wieser 2000; Mayr et al. 2003b). Accordingly, Mayr et al. (2003b) reported that embolism is rare in stone pines growing at the timberline, indicating sufficient safety margins (Choat et al. 2012; Johnson et al. 2012) even for harsh alpine winter conditions (Mayr et al. 2003a, b; Mayr and Zublasing 2010). Detailed information of stem and root *Ψ* values would be necessary to analyze elevational trends in safety margins.

Resistance to drought-induced embolism is also known to be correlated with cell-wall reinforcement (Sperry and Tyree 1990; Sperry et al. 1994; Hacke and Sperry 2001; Hacke et al. 2001). The thickness-to-span ratio [(*t/b*)^2^] indicates the resistance of a double wall to bending stress due to a pressure gradient between adjacent conduits (Hacke et al. 2001; Sperry 2003). Hacke et al. (2001, 2004) and Hacke and Jansen (2009) demonstrated that minimum values of tracheid wall thickness and wood density scale with embolism resistance in roots and stems of Pinaceae and Cupressaceae but that the (*t/b*)^2^ of stems was often greater than expected. High (*t/b*)^2^ in stems is probably based on mechanical requirements, as the aboveground axis system has to withstand gravity and wind. Accordingly, our stem samples (Table 2) showed higher (*t/b*)^2^ values than those reported in Hacke and Sperry (2001) and Hacke et al. (2004). In our study, stem (*t/b*)^2^ showed an increase with elevation (Fig. 5), which was also demonstrated in Mayr et al. (2006) and is probably related to increasing mechanical stress (wind, snow and ice loads) at higher elevation. In contrast, root (*t/b*)^2^ fit with the general (*t/b*)^2^ versus *Ψ*_50_ relationship reported for conifers (Hacke et al. 2004), and root (*t/b*)^2^ decreased with elevation (Table 2; Fig. 5). This decrease might be caused by limited cell-wall growth at higher elevation (Tranquillini 1979). Roots are predominantly exposed to tension stress so that adaptations in cell-wall reinforcement are probably less important in this plant organ than in stems (Hacke and Jansen 2009).

## Conclusion

Physiological limitation can only partly explain the observed root hydraulic patterns along the elevational transect of *P. cembra* trees up to the timberline. In our study, limitation at the root system level and at the xylem anatomical level corresponded to a trend of reduced hydraulic efficiency at high elevation, but no corresponding effect on hydraulic safety was observed, indicating that sufficient embolism resistance is important for trees at the timberline to overcome higher stress intensities. However, future studies on elevational changes in pit architecture are needed to better understand the underlying limitations and adaptations in both hydraulic efficiency and safety.
